# *NLRP1* mutations cause autoinflammatory diseases in human

**DOI:** 10.1186/1546-0096-13-S1-O22

**Published:** 2015-09-28

**Authors:** S Grandemange, E Sanchez, P Louis-Plence, C Rittore, JC Reed, I Touitou, D Geneviève

**Affiliations:** 1 INSERM, U1183, Montpellier, France; 2 CHRU, Rare and autoinflammatory diseases laboratory, Montpellier, France; 3 CHRU, Medical Genetics Department, Montpellier, France; 4 Sanford-Burnham Medical Research Institute, La Jolla, CA, USA; 5 University, Montpellier, France

## Introduction

Inflammation is a vital and complex process in response to diverse tissue damaging stimuli such as trauma, injury and pathogen. NLRP1, NLRP3 and NLRC4 belonging to the intracellular proteins Nod like receptor family, are capable of sensing the inflammatory inducers and trigger the assembly of a large complex called the inflammasome. By inducing the caspase-1 activation, inflammasome plays a crucial role in the release of IL-1β and IL-18, two critical cytokines of the initial steps of inflammatory responsesand, in some cases, the induction of a pro-inflammatory cell death called pyroptosis.

Whereas mutations in *NLRP3* and *NLRC4* have been linked to two rare monogenic systemic autoinflammatory diseases (SAIDs), several polymorphisms in the *NLRP1* gene have been associated extensively to an increased risk of autoimmune disorders (e.g. vitiligo, psoriasis, type 1 diabetes, and rheumatoid arthritis). We identified for the first time two distinct *NLRP1* mutations in patients displaying a novel systemic autoinflammatory disease (SAID) and a novel syndrome combining autoinflammation and autoimmunity.

## Objectives

The aim of our study was to unravel how mutation in NLRP1 impaired its function and triggered autoinflammation.

## Materials and methods

Peripheral blood mononuclear cells from patients and healthy donors were analyzed to identify the immunologic components involved in these novel diseases, using flow cytometry and *ex vivo* NLRP1 inflammasome stimulation. The pathogenic effect of the *NLRP1* mutations in inflammation was investigated using *in vitro* functional assays in transfected HEK293T.

## Results

An immunophenotyping in one patient revealed high numbers of granulocytes, CD64+ neutrophils, NK cells and immature blood B cells (CD20+CD27-CD38highCD24high). The level of caspase-1 in serum samples from patients was increased as compared to controls and unaffected parents. Moreover, patient's cells displayed constitutive production of IL-1β. Functional studies in HEK293T are ongoing in an attempt to confirm constitutive activation of the NLRP1 inflammasome suggested by our results on patient's samples.

## Conclusion

We demonstrated for the first time that two mutations in the *NLRP1* gene are involved in autoinflammation in human. Our data, combined to the literature, highlight the pleomorphic roles of NLRP1 in inflammation and immunity.

